# CNV-CH: A Convex Hull Based Segmentation Approach to Detect Copy Number Variations (CNV) Using Next-Generation Sequencing Data

**DOI:** 10.1371/journal.pone.0135895

**Published:** 2015-08-20

**Authors:** Rituparna Sinha, Sandip Samaddar, Rajat K. De

**Affiliations:** 1 Department of Information Technology, Heritage Institute Of Technology, Kolkata, West Bengal, India; 2 Department of Computer Science and Engineering, Heritage Institute Of Technology, Kolkata, West Bengal, India; 3 Machine Intelligence Unit, Indian Statistical Institute, Kolkata, West Bengal, India; Emory University School Of Medicine, UNITED STATES

## Abstract

Copy number variation (CNV) is a form of structural alteration in the mammalian DNA sequence, which are associated with many complex neurological diseases as well as cancer. The development of next generation sequencing (NGS) technology provides us a new dimension towards detection of genomic locations with copy number variations. Here we develop an algorithm for detecting CNVs, which is based on depth of coverage data generated by NGS technology. In this work, we have used a novel way to represent the read count data as a two dimensional geometrical point. A key aspect of detecting the regions with CNVs, is to devise a proper segmentation algorithm that will distinguish the genomic locations having a significant difference in read count data. We have designed a new segmentation approach in this context, using convex hull algorithm on the geometrical representation of read count data. To our knowledge, most algorithms have used a single distribution model of read count data, but here in our approach, we have considered the read count data to follow two different distribution models independently, which adds to the robustness of detection of CNVs. In addition, our algorithm calls CNVs based on the multiple sample analysis approach resulting in a low false discovery rate with high precision.

## Introduction

Copy number variation is a type of genomic structural alteration, which is caused by either duplication (or insertion) of a large genomic segment multiple times, or may be characterized by the deletion of a large DNA segment. The size of the region getting duplicated, deleted or inserted ranges from kilobases (kb) to megabases (mb) [1]. These copy number variations can be found in human as well as other mammals [2]. The human genome is composed of more than 25000 genes, and it was known earlier that a gene is always present in copies of two. But recent studies have proven that a gene can be present in one, two, three or more numbers of copies or it may happen that the whole gene is deleted. This happens due to insertion or deletion of large chunks of DNA segment, which may encompass genes causing changes in their copy number, and thereby leading to dosage imbalance. CNVs can have a vital impact on human health. It is associated with some complex diseases related to neurological disorders, including autism spectrum disorder (ASD) [3], schizophrenia [4], and also associated with some cancers [5]. Duplications of many oncogenes and deletions of tumor suppressor genes may lead to the onset of a cancer [6]. However, the presence of this form of structural variations (CNVs) does not always relate to diseases, rather it may also be present in some healthy individuals. Hence, the detection of CNV regions is an important task.

In earlier days, fluorescence in situ hybridization (FISH) [7] and array comparative genomic hybridization (aCGH) [8] based techniques were used to detect CNVs. These techniques suffered from low resolution and noise due to hybridization, and detection of CNV breakpoints (starting and ending position) was also not very precise [9]. The demand for low cost sequencing has led to the development of next generation sequencing (NGS) technology that parallelizes the sequencing process by generating millions of short reads, involving low cost and time [10]. NGS provides us a new dimension towards detection of CNVs with high coverage, high resolution, and a platform for efficiently detecting novel and rare CNVs. These NGS based algorithms use DNA sequence reads, and map them against a reference genome sequence to detect any kind of variations.

NGS based CNV detection methods are mainly divided into two categories: paired end mapping (PEM) based approaches [11] and depth of coverage (DOC) based approaches [12]. PEM based methods use paired end reads. The pair ends of the sample genome are mapped against the reference genome, and the distance between the paired ends of the sample and that of reference is calculated. If the two distances vary significantly, then the presence of deletion or insertion is there in the sample. PEM based methods have limitations of finding insertions, deletions of larger sizes [11]. These methods also have limitations in detecting regions having segmental duplications. DOC methods are more commonly used in CNV detections. These methods first track the alignment of short reads to non overlapping windows (bins) or sliding windows of the reference sequence, resulting in read count or read depth data. Unlike PEM based methods, DOC based algorithms can detect CNVs of larger sizes, detect CNVs in the complex genomic region, and can also estimate the exact copy number of genomic regions.

Some of the read depth based approaches include EWT (Event-wise Testing) introduced by Yoon [13], which uses the high throughput sequence data and filters out reads of low quality. Reads mapping to 100 bp window are counted and then GC-corrected normalized read count information is taken into account. The modified read count information is converted to z-scores and uses a probabilistic model to combine windows to maximize the score. JointSLM [14] extends the idea of EWT, and uses a multiple sample analysis, based on a statistical analysis using hidden Markov model. CNVeM [15] uses a probabilistic model and uses expectation maximization algorithm to detect CNV of individual samples. CNVeM identifies breakpoints with high resolution as it detects CNVs at the nucleotide level. cnMOPS [9] detects CNVs across multiple samples using a mixture of Poisson model. Another multiple sample analysis method is correlation matrix diagonal segmentation (CMDs) [16]. CMDs is based on a between-chromosomal site correlation analysis and is used to detect recurrent copy number aberrations (RCNA). As the input, it takes the copy numbers or CN data of genomic regions, and based on correlation coefficients, diagonal transformation is performed [17].

As multiple sample analysis improves performance and causes low false discovery rate [9] with high precision, we are motivated to work on multi sample analysis approach. In our work, we have detected human genomic locations with copy number variations by using a cross sample analysis approach. Our algorithm, CNV-CH, works on NGS data where millions of short reads are generated in parallel, from the DNA sequence of a particular chromosome. CNV-CH is based on the depth of coverage data where the transformation of read count information into geometrical points is done based on some statistical measures. To our knowledge, most of the existing algorithms assume the read count data to follow a particular statistical distribution, whereas we have considered the distribution of read count data to follow two different distributions independently. It adds robustness against the error which may arise from incorrect assumption of a particular statistical distribution model. In addition, as there exists variability in read count data, an appropriate smoothing technique at each and every base pair or locus across the genome, is required as a part of data preprocessing. The variability in read count data occurs due to mappability bias and a certain level of non-uniform read generation in NGS technology. The present method CNV-CH has considered this issue by using an exponential smoothing approach, where the mappability score has been used to determine the exponential factor.

Another important aspect of CNV-CH is the deployment of a new segmentation algorithm to segment genomic regions depicting copy number changes. The segmentation algorithm is based on the notion of a convex hull algorithm applied over two dimensional geometric points, where each point represents two statistical measures. Based on the area of the convex hull, we are able to detect genomic regions having copy number variations. The method performs better compared to some other single sample analysis approaches, since it detects CNVs with high precision and low false positive rate. Moreover, the present algorithm detects CNV breakpoints with high computational efficiency. In this work we performed simulations considering all possible experimental conditions, where each condition is a combination of values corresponding to the coverage, variant length, copy number and number of test samples. As a result of which 360 experimental conditions were generated, and under each of these conditions, we conducted 100 trials, as described in the Results section. CNV-CH has also analyzed the real sequencing data of chromosome 20 of multiple human individuals. These human genome sequences are based on deep coverage whole genome sequencing data. Each detected region was validated with the variants listed in the Database of Genomic Variants (DGV) (http://www.dgv.org). The CNV regions detected by our method (CNV-CH), were mostly larger than 10 kbp of length. Moreover, our algorithm has also detected variants as small as 0.6 kbps.

## Results

We demonstrate the effectiveness of our algorithm on both simulated and real data along with its superior performance over four publicly available methods in detecting CNVs. These publicly available methods are EWT [13], cnMOPS [9], CMDs [16] and CNV-TV [18].

As CNV-CH involves a cross sample analytical tool for NGS data, it needs high computational resource in terms of memory. We implemented our algorithm using a 64 bit machine with 16 GB memory. The complexity of the algorithm was found to be *O*(*nklogk*) where *k* is the total number of samples and *n* is the number of 100bp bins. As cross sample analysis has been found to produce effective results with 6–10 samples, hence the complexity *O*(*nklogk*) has almost become equal to *O*(*n*) as *klogk* can be approximated as a constant term, where, 6 ≤ *k* ≤ 10.

### Real Datasets considered in the present study

In our study, we considered chromosome 20 of human genome sequence data, which were based on deep coverage whole genome DNA sequencing on two family trios in the CEPH Utah and HapMap sample collections (http://www.1000genomes.org). All sequence reads were mapped against the NCBI build 36.3 human genome reference sequence. These reads were present in a compressed binary alignment file, i.e., as a “.bam” file obtained from (ftp://ftp.1000genomes.ebi.ac.uk/vol1/ftp/) or (ftp://ftp-trace.ncbi.nih.gov/1000genomes/ftp/). The files can also be obtained from (http://www.1000genomes.org/data). The samples were CEU trio female of European ancestry (NA12878, NA12892), and YRI females (NA19240, NA19238), and a CEU trio male of European ancestry (NA12891), and an YRI male (NA19239). We also worked on low coverage sequencing data. The samples included were CEU trio female of European ancestry (NA12878, NA12751, NA07037, NA12763, NA12813, NA12718, NA12828), YRI males (NA18501, NA18910), CEU trio male (NA12272, NA12286) and a CHB Han Chinese female (NA18565). Each of these samples was sequenced as a part of the pilot project of the 1000 genomes Project (http://www.1000genomes.org) under the sequencing platform of Illumina. We have validated the CNV’s detected by CNV-CH with the CNV’s listed in the database of genomic variants(DGV) (http://www.dgv.org).

In the simulation study, we considered a human reference sequence of chromosome 20 obtained from the site (ftp://hgdownload.cse.ucsc.edu/goldenPath/hg19/chromosomes/) or it can also be obtained from (http://hgdownload.cse.ucsc.edu/downloads.html). The simulated data generation is described in Analysis of simulated data section.

### Analysis of simulated data

In this study, we took a human DNA sequence of chromosome 20 and processed it as follows. All the N’s (unknown base) was first removed from this sequence, reducing the file size from 61.3MB to 57.8MB. We also removed all regions which are already listed in DGV (http://dgv.tcag.ca/dgv/app/home) as known variants. From this sequence, we randomly chose 10 disjoint segments of size 2MB each. Now, out of these 10 segments, 1 random segment was selected in each trial, acting as the reference genome, and another random segment was selected for simulating deletion event as described later.

**This simulation study was performed by manipulating the following experimental conditions:**

- Coverage (*C*), representing the average number of times a given base has been sequenced, by independent reads, where *C* was chosen from 2*X*, 10*X*, 15*X* or 30*X*;
- Variant length (*V*), being the length (in bp) of the genomic subsequence that was duplicated or deleted reflecting copy alterations, where *V* was chosen from 1kb, 2kb, 3kb, 4kb, 5kb or 6kb;
- Copy number (*Z*), denoting the number of times a subsequence of length *V* has been duplicated or deleted, where the value of *Z* was chosen from 0, 1, 3, 4 or 5;
- Number of test samples (*n*), selected from 10, 20 or 50; and
- Position (*P*), representing the genomic coordinate where a copy variation has been introduced.

We considered all possible experimental conditions, where each condition is a combination of values corresponding to the coverage, variant length, copy number and number of test samples, as a result of which 360 experimental conditions were generated. Under each of these conditions, we conducted 100 trials, and in each trial the following was done.

In order to simulate a diploid genome, the control genome was generated from the reference genome, and was concatenated with its copy. Thus the size of the control genome became 4MB. Now, *n* test samples were also generated from the diploid control genome, wherein each individual test sample, we introduced copy number variation in a randomly selected position *P*. According to the experimental condition corresponding to a trial, a subsequence from position *P* to *P* + *V* was duplicated *Z* − 2 times, when *Z* > 2 (copy gain). A deletion event, when *Z* < 2, was simulated by choosing a subsequence from *P* to *P* + *V* from the second 2MB segment and inserting it into the reference genome at the same position *P*. As this subsequence would be present in the reference genome, but absent in test sample, it would simulate a deletion event (copy number 0) in that position. In the case of deletion with copy number 1, the above subsequence was inserted in the reference genome (at position *P*) as well as in the test genome at either position *P* or *P* + 2MB. We also introduced mutation of 1bp randomly at an interval of 300bp in each test sample. The decision to implant a copy number variation by the above process in a test sample, was made by a coin tossing event.

Reads were generated from each of the test samples, where the length of each read was set to 36bp, to keep parity with the size of short reads generated by Illumina technology. The read generation process was done by sampling reads with a probability *r*, which reflects GC-content of the read. The values of *r*, used in read sampling process, were determined from a unimodal [19] probability distribution function, obtained using the median GC-count of all possible 36-mers of chromosome 20 sequence (human reference hg19). Thus, it introduces GC-bias in short read generation process. This sampling of read was repeated till a desired level of coverage *C* was achieved. Now, reads were aligned to 100bp non-overlapping bins of the reference sequence. Since, the reads being originated from a repeat rich region of the test genome, might have more than one possible optimal alignment in the reference, hence, aligning them according to our method will introduce mappability bias in read count data. This mappability bias was corrected by the process discussed in Materials and Methods section. Finally, for each of these 20972 bins of a test sample, we obtained the corresponding adjusted read count data.

**Fig 1:**
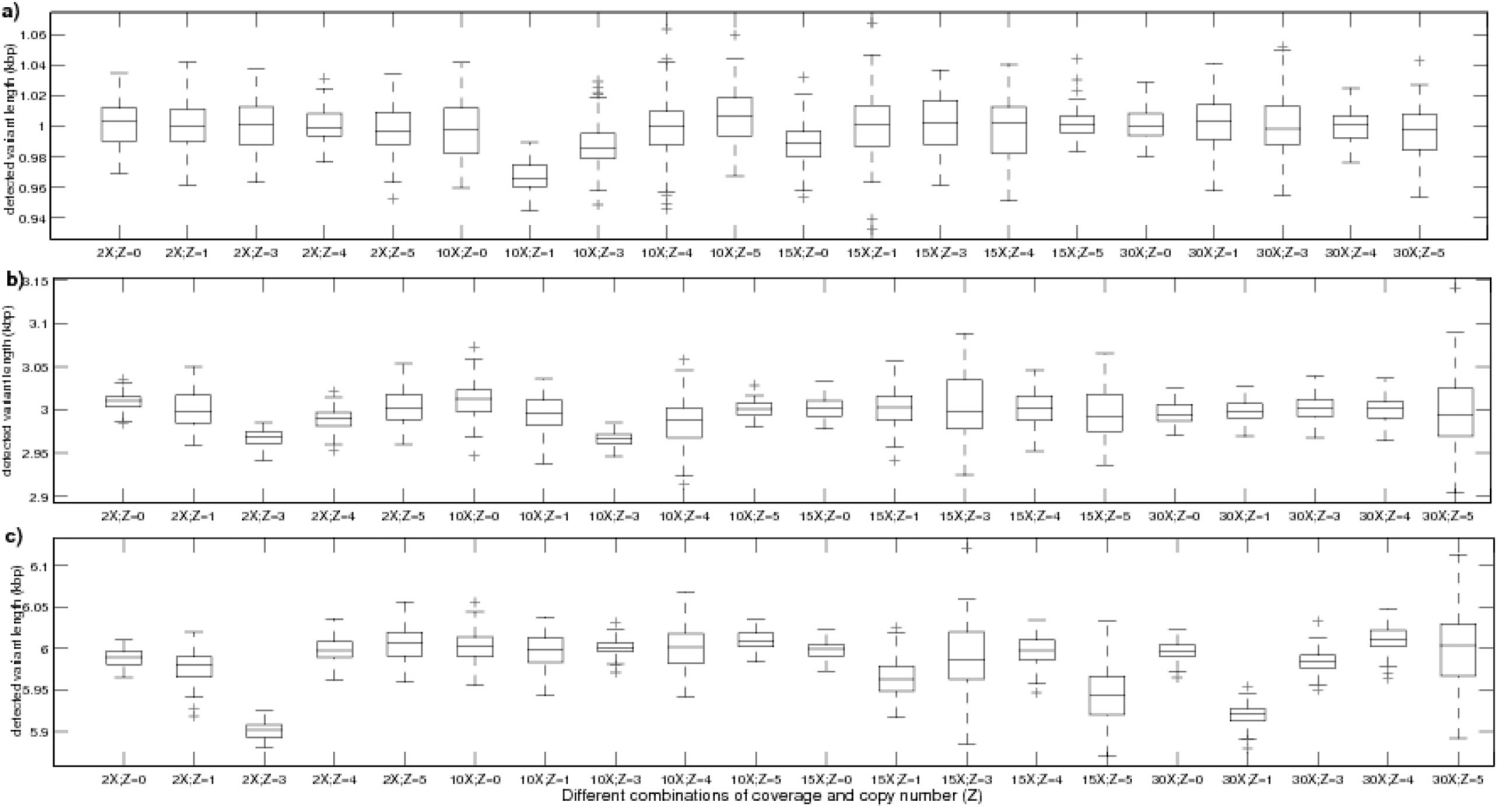
The box plot based analysis of the detected length of variations, obtained from simulation results on the implanted CNV length of 1kbp, 3kbp and 6kbp with 10 samples. (a) represents the length (kbp) of the regions with variations, detected by CNV-CH in a set of 100 trials, for all combinations of coverage (C) and copy number (Z), and at fixed implanted variant length of 1kbp. The horizontal solid line within a box indicates the median variant length detected with a set of 100 trials, and the +’s indicate the outliers. (b) represents the length (kbp) of the regions with variations, detected by CNV-CH in a set of 100 trials, for all combinations of coverage (C) and copy number (Z), and at fixed implanted variant length of 3kbp, whereas in (c), the implanted variant length is 6kbp.

The outcome of the simulations, performed under a fixed single copy length and fixed number of samples with varying coverage level and copy number, is depicted using box plots, as represented in Fig 1. Each outcome is the single copy length of the detected region, having variation in copy number with respect to the reference sample. Each experimental condition was set by altering the coverage (C) and copy number (Z), and with a fixed single-copy variant length of 1kbp, 3Kbp and 6Kbp as depicted in Fig 1(a)–1(c) respectively. Trials under each experimental condition, represented by Fig 1, were performed with 10 samples (i.e., *n* = 10). Thus, each of the figures show 20 benchmark (experimental) conditions, where the outcome of 100 trials, performed under each such condition, is represented by a box in the figure. For instance, the rightmost box of Fig 1(a) shows the range of variant length detected by CNV-CH in 100 simulated trials, at 30*X* read coverage and copy number 5. It is observed that the median value of the detected variant lengths of each box (corresponding to a set of 100 trials performed under one of the 20 conditions) is very close to the actual implanted single copy variant length of 1Kbp. In addition, it is also observed that the lower edge of the box, i.e., the lower quartile *q*1 (25 percentile) and the upper edge of the box, i.e., the upper quartile *q*3 (75 percentile) are also between 0.98kbp and 1.02kbp, except at 10X coverage with copy number 1 (deletion), the value of *q*1 is around 0.96Kbp. Similarly, in Fig 1(b) and 1(c), it is observed that the median value in each box (corresponding to trials conducted under one of the 20 conditions) is very close to the actual implanted single-copy variant length of 3Kbp and 6Kbp. Fig 1(b) reflects that the lower quartile *q*1 and the upper quartile *q*3 were bound between 2.95 and 3.05, whereas in Fig 1(c), the quartiles were between 5.89 and 6.04.

The outcome of the trials under each condition was treated as outliers (‘+’ mark), if they were larger than *q*3 + 1.5(*q*3 − *q*1) or smaller than *q*1 − 1.5(*q*3 − *q*1), where the factor 1.5 corresponds to ±2.7*σ* (*σ* being the standard deviation of the outcome) and 99.3 coverage approximately, under an assumption that the outcomes of the trials conducted under a particular condition are normally distributed. It is observed in Fig 1(a) that no outlier exists among any outcome of trials, conducted under 10 different conditions for 1Kbp single-copy variant length, while only one outlier is observed, in each set of outcomes under the other 5 conditions. On the other hand, trials under the remaining 5 conditions, revealed at most 5 outliers, where the maximum number of outliers occurred under 10*X* coverage, with copy number 4. Fig 1(b) shows no outlier corresponding to 14 different conditions under 3Kbp single copy variant length. Only one or two outliers was/were equally observed in trials conducted under the remaining 6 conditions. Fig 1(c) also revealed the presence of no outlier under 11 conditions with fixed single-copy variant length of 6Kbp.

**Fig 2:**
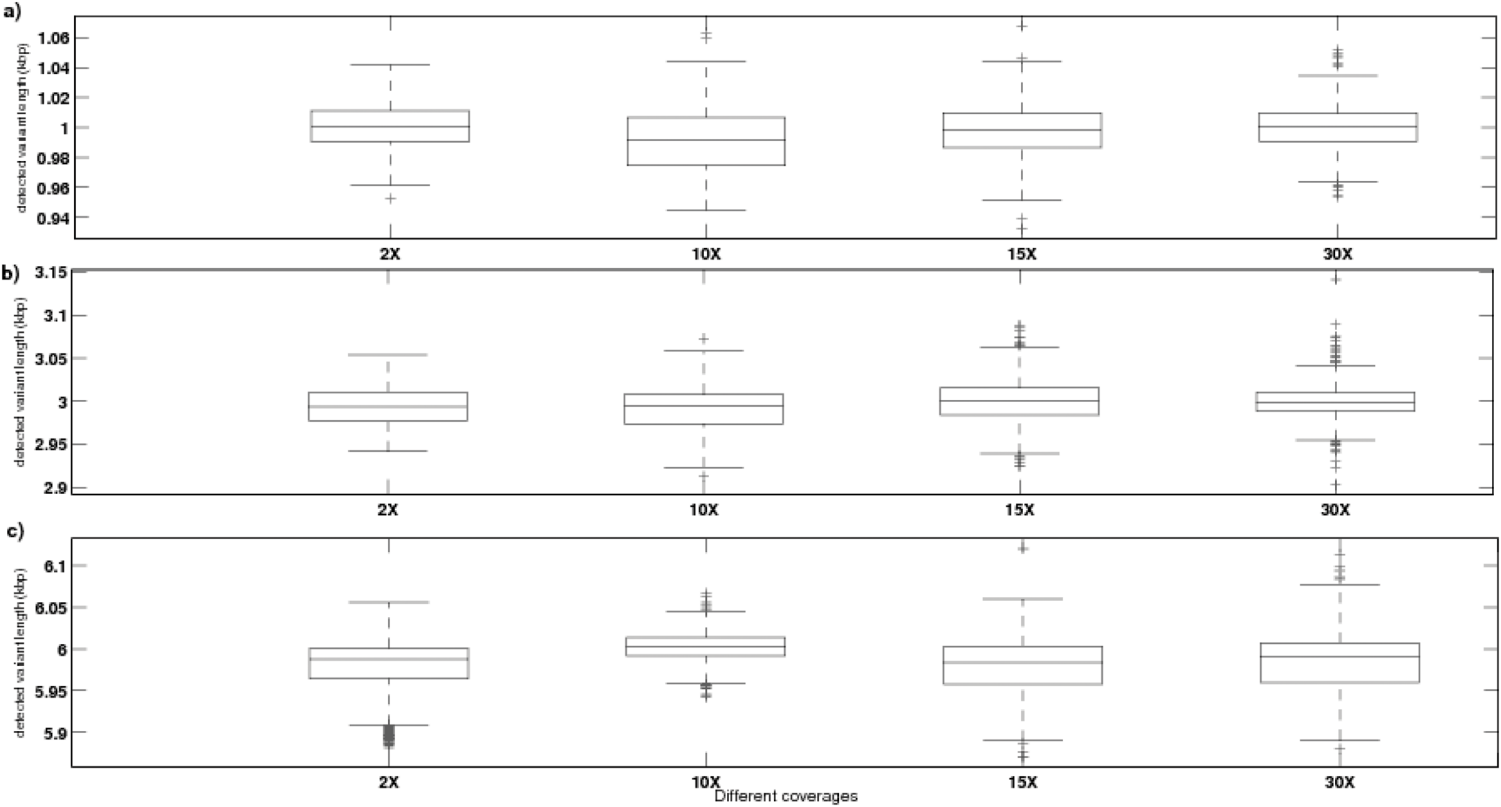
The box plot based analysis of detected length of variations, obtained from simulation conducted at each coverage level (C) (2*X*, 10*X*, 15*X* or 20*X*), with all possible copy number variations (Z) (0, 1, 3, 4 or 5) and at a fixed single-copy variant length of 1Kbp, 3Kbp and 6Kbp as depicted in (a), (b) and (c) respectively, with 10 samples.

Fig 2(a)–2(c) depict the outcome of trials conducted at each coverage level (C), with all possible copy number variations (Z) and at a fixed single-copy variant length of 1Kbp, 3Kbp and 6Kbp respectively, on 10 samples. Here, each outcome represents the single copy length of the region detected by CNV-CH, having any variation in copy number (with respect to the reference sample). Each box in the figure represents the range of outcomes obtained from a set of 500 trials, with a fixed single-copy length and coverage level. It is observed that there exist no outliers at low coverage level of 2X, with all possible copy numbers Z and at single-copy variant length of 3Kbp, while only one outlier was found with copy length of 1Kbp, as shown in Fig 2(a) and 2(b). But at 2*X* coverage with single-copy variant length of 6Kbp, about 7 outliers exist, as shown in Fig 2(c). At 10*X* coverage level, we observed presence of only 2 outliers at each single-copy variant length of 1Kbp and 3Kbp, for all possible copy numbers Z, as represented in Fig 2(a) and 2(b). At coverage levels of 15*X* and 30*X*, with single-copy variant length of 1Kbp, 3Kbp and 6Kbp, we observed 4–14 outliers as seen in Fig 1(a)–1(c) respectively.

**Fig 3:**
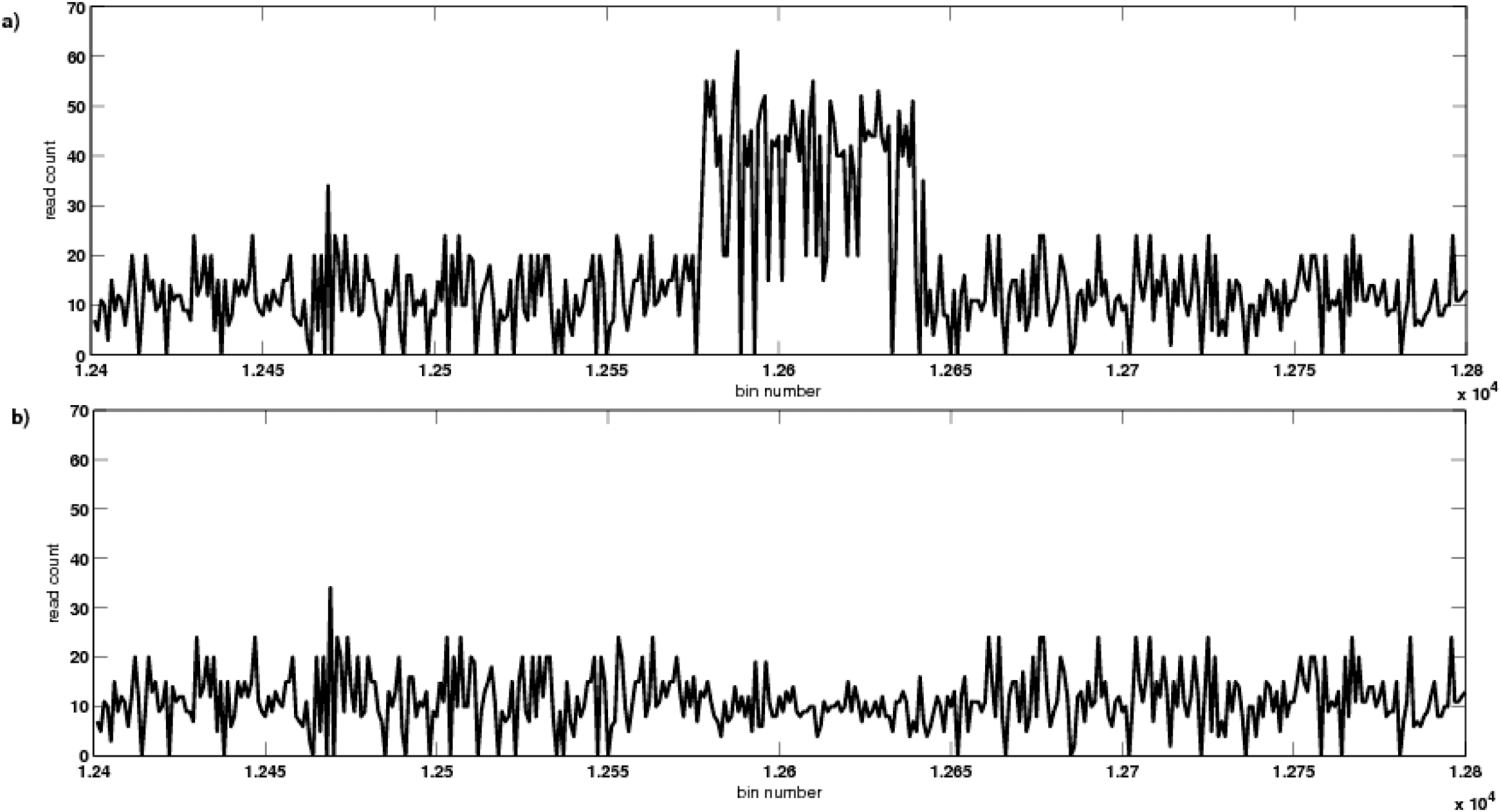
An instance of GC-corrected read count data, corresponding to the genomic region 1240000bp–1280000bp, of 2 test samples, as represented in (a) and (b). The copy number variation as duplication, was introduced in the genomic segment 1257695bp–1263695bp as represented in (a). The other sample has no variation in this region, as represented in (b).

**Fig 4:**
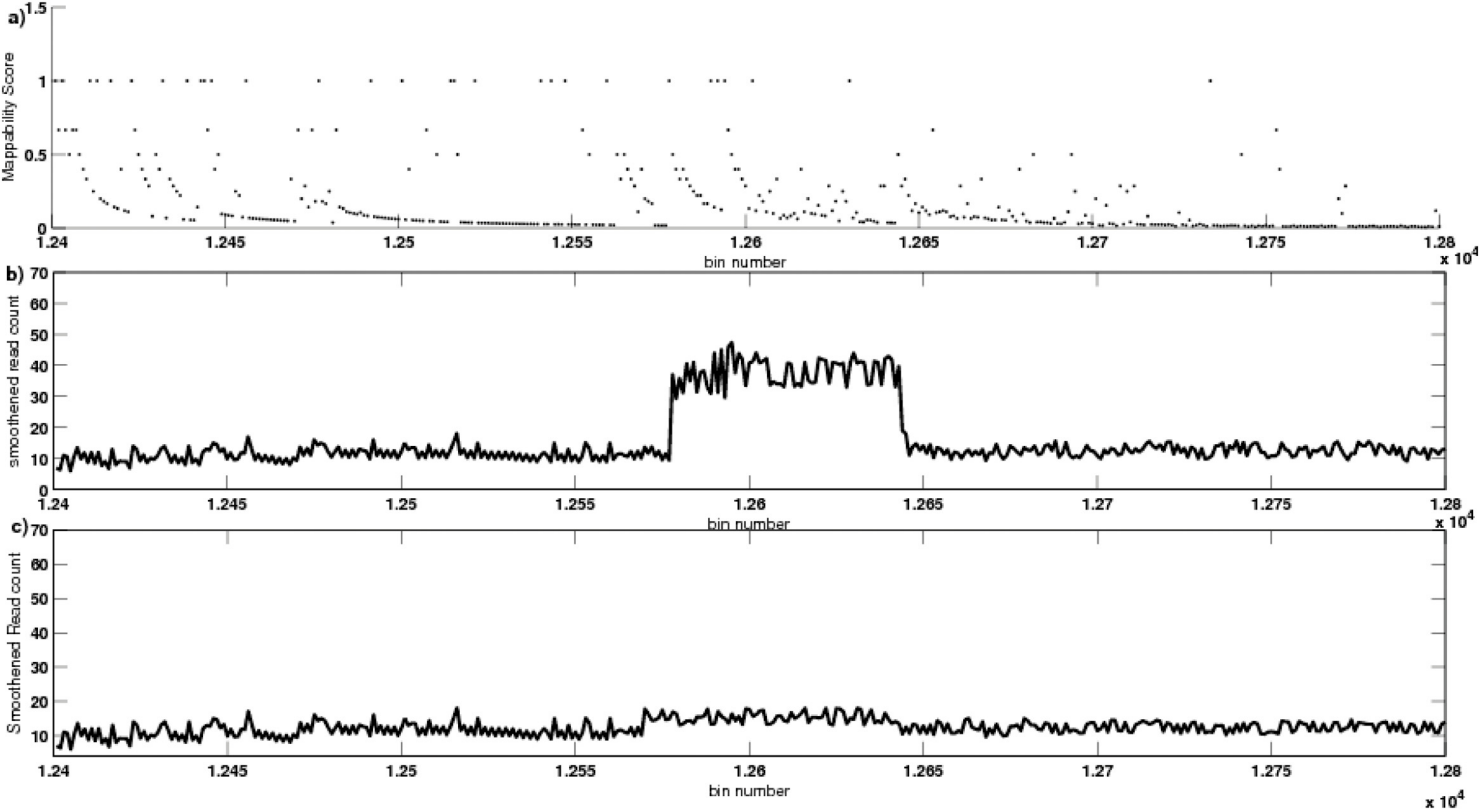
The mappability score of the reference sequence and its correction, of a trial instance. (a). The mappability score varies widely in [0, 1], corresponding to high to low repeat rich region in the genome, respectively. (b) and (c), depicts the smoothed read count signal obtained after removing the mappability bias from the GC-corrected read count data.

**Fig 5:**
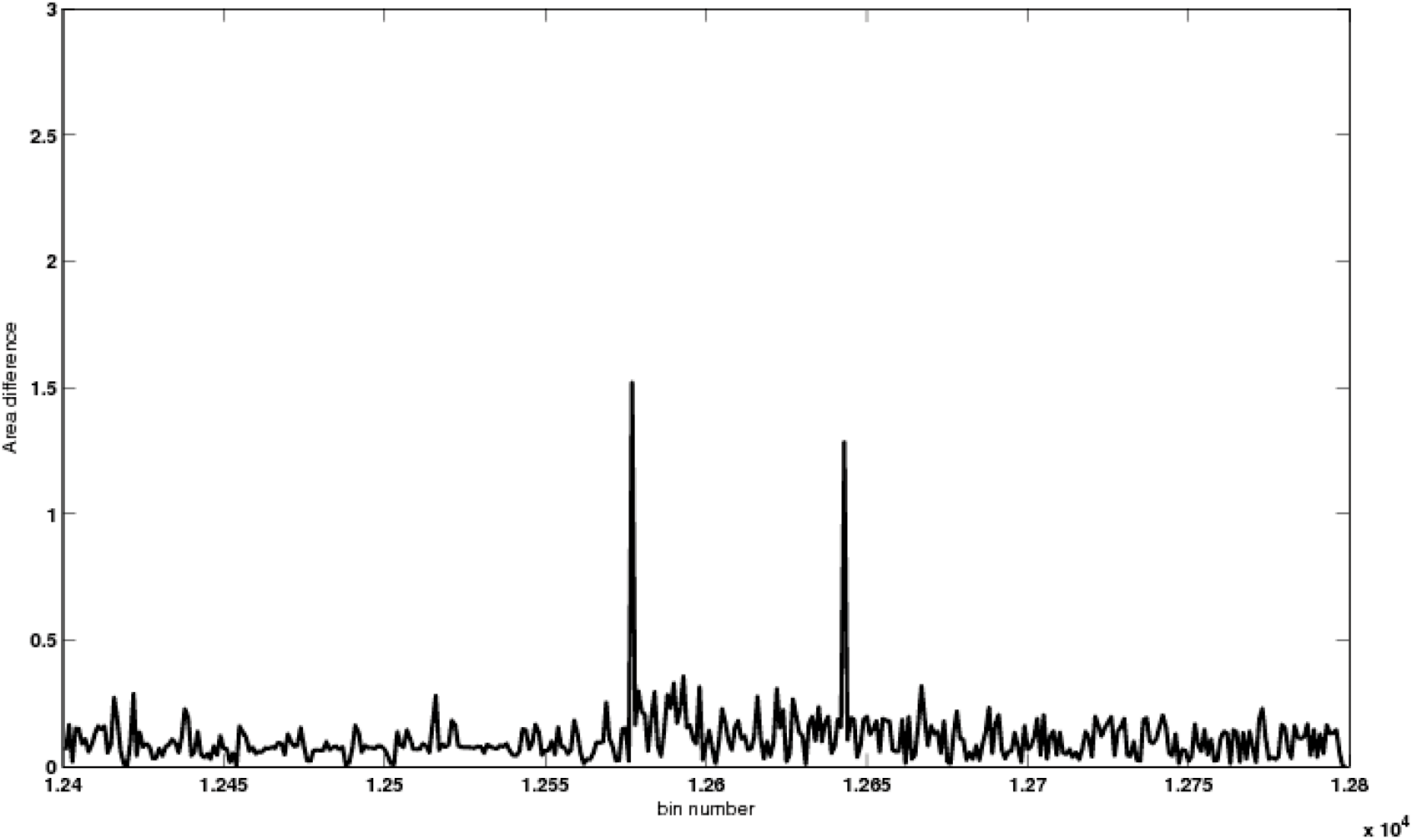
The breakpoints achieved across the genomic region 1240000bp–1280000bp. The start bin and end bin of the genomic region having copy number changes, has a high value of △.

Fig 3(a) and 3(b) depict an instance of GC-corrected read depth of 2 samples in the region 1240000bp–1280000bp, taken from one of the trials at a coverage level of 15*X*. A CNV using single-copy variant length of 6Kbp was introduced, by performing duplication of the genomic coordinate 1257695bp–1263695bp, to obtain copy number 3. Fig 3(a) shows the read count data (after removing GC-bias) of one of the samples where this variation was introduced. Fig 3(b) shows the GC-corrected read count data of the other sample in the same instance of trial, where this variation was not introduced, based on an unbiased coin toss event as mentioned earlier. These GC-corrected read count data of the two samples were suffering from mappability bias, as observed in the figure. To remove this mappability bias, we applied a new technique, using exponential smoothing and mappability score, as discussed in Materials and Methods section. This mappability score of the reference sequence of a trial instance is represented in Fig 4(a). It is also observed that the mappability score varies widely in [0, 1], corresponding to high to low repeat rich region in the genome, respectively. Fig 4(b) and 4(c) depict the smoothed read count signal obtained after removing the mappability bias from the GC-corrected read count data. Fig 5 represents the difference in area, i.e., the value of △ (in Eq 5) obtained by our algorithm, where the threshold value was considered as 1.0. It is observed that bin 12578 was having a very high value of △. Thus, it was considered as a starting boundary (break point) of an abnormal genomic region. Again bin 12637 was also having an increase in the value of △, thereby, this bin was considered as the end boundary. Thus, the region with genomic coordinate 1257701bp–1263700bp, was detected by CNV-CH as a region having copy number variations in one of the trial instances.

### Analysis of real data

CNV-CH analyzed the real sequencing data of chromosome 20 of multiple individuals. Each detected region was validated with the variants listed in the Database of Genomic Variants (DGV). The performance of CNV-CH on real sequencing data was analyzed on different combinations of high coverage and low coverage real data of chromosome 20. In one of the execution instances, we considered 4 high coverage data samples and two low coverage samples. In this instance, among 6 high coverage samples NA12878 and NA19240 were excluded, which are the offsprings of CEO pair—NA12891 and NA12892, and YRI pair—NA19238 and NA19239 respectively. Thus, 4 parent samples were combined with 2 low coverage samples, NA07037 and NA18501 in the execution instance 1. The results obtained from execution instance 1 is shown in Table 1. In the second execution instance, we included both the offsprings NA12878 and NA19240, excluding all four parents of instance 1, and combined them with low coverage samples NA12813, NA07037, NA12751 and NA18910. In the third instance, CNV-CH was executed using all the high and low coverage data. CNV-CH detected the same set of variants in NA12878 for both the execution instances 2 and 3. Similarly, in NA19240, CNV-CH again identified the same set of variants for both the execution instances. It indicates that the performance of CNV-CH is independent of hereditary relationship among the samples. We also observed that in NA12878, out of 378 detected CNVs, 58 variants were common with its parent NA12891, and 64 variants were common with the other parent NA12892. Similarly, NA19240 had 128 variants common with one of its parent NA19238, and 84 variants were common with its other parent NA19239.

**Table 1:** Results obtained by CNV-CH for the execution instance 1 of real data.

**Samples**	**Detected Variants**	**Validated Variants**
NA12891	308	283
NA12892	202	181
NA19238	518	481
NA19239	624	529
NA07037	184	172
NA18501	388	324

The first column represents the samples taken into consideration for the execution instance 1. The second column of the table shows the total number of variants detected by CNV-CH, and the third column shows the number of detected variants that has been validated.

An instance of the detected CNVs (as found in execution 3), across all the samples of a real data set in the region 26200000bp and 30000000bp, is listed in Table 2 (see supplementary for all the regions with variations, detected by CNV-CH in human chromosome 20). The first two columns of the table show the genomic coordinates (start and end) of the CNVs that were detected by our algorithm. The third and fourth columns of the table show the overlapped region, i.e., the genomic coordinates of each of our detected CNVs that overlap with the genomic coordinates of the CNVs that are listed in the Database of Genomic Variants (DGV). The bins 261816–262217, 262295–262418, 262421–262450 and 297454–297733 were affected by variation in almost all the samples, whereas the bins in which the CNVs were detected in few samples are 295173–295367, 295401–295453, 295456–295515 and 295623–295726. CNVs in the region 29577292bp–29596682bp and the region 29600079bp–29605289bp were present in the samples NA12878, NA12891, NA12892 and NA19238. CNVs in the region 29605587bp–29611484bp were present in only NA12878 and NA12892, whereas the region 29622290bp–29632579bp were detected only in samples NA12878, NA12891 and NA12892. In chromosome 20 of the tested samples, the CNV regions detected by our method, are mostly more than 10 kbp in length. However, smaller variants were also detected by CNV-CH, where the length was as small as 0.6 kbp.

**Table 2:** Summary of the CNVs identified in the region 26200000bp–30000000bp, and their validation with Database of Genomic Variants (DGV).

**Detected Start Coordinate (bp)**	**Detected Stop Coordinate (bp)**	**Validated Start Coordinate (bp)**	**Validated Stop Coordinate (bp)**
26241583	26281679	26249913	26250767
26289488	26301786	26298284	26301786
26302082	26304977	26302082	26302232
29577292	29596682	29577292	29596682
29600079	29605289	29600079	29605289
29605587	29611484	29605587	29611484
29622290	29632579	29622290	29632579
29805380	29833288	29805380	29832573

The first two columns of the table show the genomic coordinates (start and end) of the CNVs that were detected by our algorithm. The third and fourth columns of the table show the overlap region, i.e., the genomic coordinates of each of our detected CNVs that has overlapped with the genomic coordinates of the CNVs listed in DGV.

### Methods compared in our work

We compared the performance of CNV-CH with four existing algorithms used for analysis of copy number variations. The algorithms compared with CNV-CH were EWT, CNV-TV, CMDs and cnMOPS. Among them, cnMOPS, EWT and CNV-TV used NGS data. In addition, these algorithms detect CNVs by analysis of read count data.

EWT [13] implements a read depth based CNV detection tool, called RDXplorer, which uses event wise testing, assuming a normal distribution for the read count data. We implemented EWT considering *z*-score of GC-corrected read-count data and a nominal false positive level of 0.05. Detection of an event was associated with the occurrence of significant high or low read count data among a set of consecutive windows. The size of each such consecutive window was fixed to 100bp. Then merging of small events was performed using Z-test with a significance level of 10^−6^ for minimizing false positive detections. We assumed an abnormal event to be small, if it spans less than 5 bins or windows, i.e., the minimum size of genomic region with CNV, was set to 500bp.

Implementation of cnMOPS [9] was done for comparison with CNV-CH. Like CNV-CH, it also detects CNVs by analyzing a particular genome of multiple samples, using a mixture of Poisson model. While implementing cnMOPS, the median read count was chosen as an initial estimation of the mean read count. The initial value of the parameter *α* was the same as that in the original implementation in [9]. While implementation of cnMOPS, the detected candidate segment was considered to be a CNV, if the information gain of the posterior of the parameter *α*, compared to its prior distribution (i.e., I/NI call), had a value greater than 0.6 or less than -1.0, as described in the original implementation [9].

CNV-TV [18] used total variation penalized least squares for smoothing the read depth signal. CNV-TV was implemented by using the SolveLasso function (SparseLab package of http://sparselab.stanford.edu). CMDs [16] is a multi sample based CNV analysis tool, based on the processing of microarray intensity data. We implemented CMDs on read count data by applying a correlation coefficient as a measure of similarity among adjacent chromosomal sites, as described in [16]. We considered each chromosomal site, i.e., a window or a bin of size 100 bp. As CMDs processes CN (copy number) data, we pre-estimated the copy number of each chromosomal site, and used it as an input data in the implemented CMDs algorithm.

Table 3 represents an instance of the percentage of overlap (in terms of base pair) between the CNV regions detected by CNV-CH, and all the algorithms compared with, as observed in the segment 26200000bp–30000000bp of chromosome 20. In addition, we also provided the corresponding percentage of overlap with the regions, reported in DGV, in the same chromosomal segment. Based on the CNVs listed in Table 2, the first column in Table 3 represents the coordinates of the CNVs detected by CNV-CH in the above mentioned region of chromosome 20. For a particular row, each entry shows the percentage of overlap with DGV, EWT, cnMOPS, CNV-TV and CMDs respectively. The second column of Table 3 depicts the percentage of base pair overlap with those reported in database of genomic variants (DGV). On an average 78.22 percent base pair overlap with DGV was observed in the segment 26200000bp–30000000bp of chromosome 20, which is quite good at the base pair level. The third column represents the percentage overlap obtained by EWT. The average value was found to be 87.8 percent. The fourth column denotes how much the CNVs, detected by cnMOPS, overlapped with that obtained by CNV-CH. On average, the overlap percentage was 19 percent. The fifth and sixth columns represent how much CNVs, detected by CNV-TV and CMDs, overlapped with that obtained by CNV-CH. It was observed that the outcome of CNV-TV, on an average, overlapped by 31 percent and that of CMDs by 27.5 percent. It was found that the outcome of CNV-CH overlapped maximum with that of EWT. It was also observed that cnMOPS was unable to detect many regions which our algorithm, CNV-CH, detected and validated.

**Table 3:** The overlapped percentage of base pairs of CNV regions detected by the aforesaid algorithms.

**Region Detected (bp)**	**DGV** %	**EWT** %	**cnMOPS** %	**CNV-TV** %	**CMDs** %
26241583–26281679	2.13	100	38.70	12	20.45
26289488–26301786	28.5	.05	19.68	68.38	78.01
26302082–26304977	100	100	0	1.56	34
29577292–29596682	100	100	0	23	7
29600079–29605289	100	100	0	0	17
29605587–29611484	100	100	0	1.01	45.22
29622290–29632579	100	100	0	98.12	12
29805380–29833288	95.13	96.8	85.84	43.6	6.23

The first column represents the CNVs detected by CNV-CH in the region 26200000bp–30000000bp of chromosome 20. The second column represents the corresponding percentage of overlap with the regions, reported in the Database of Genomic Variants (DGV), in the same chromosomal section. For a particular row, each entry shows the percentage of overlap of CNV-CH with DGV, EWT, cnMOPS, CNV-TV and CMDs respectively.

The quality of the output of our algorithm was evaluated and analyzed using various standard measures, like specificity, precision, sensitivity, F-Score and markedness, defined as follows. Specificity denotes the proportion of true negatives with respect to the sum of true negatives and false positives. Sensitivity denotes the proportion of correctly detected CNVs in a genome, with respect to the total number of CNVs actually present in that genome. This sensitivity is also called as true positive detection rate or recall. On the other hand, positive predictive value or precision signifies the proportion of truly detected CNVs with respect to the total number of detected CNVs (including both false and true detection). The objective of any ideal CNV detection algorithm is to optimize all these measures, but practically some of these measures adversely affect each other. Hence, tradeoff criteria between these measures has to be adopted, considering which we have taken two additional scores, viz., F-Score and markedness. F-score reflects a measure of the quality of validation of detected CNVs with respect to true CNVs, and Markedness reflects the overall quality of detected and undetected segments. F-score was evaluated using the expression (2 * *precision* * *sensitivity*)/(*precision* + *sensitivity*), and markedness was evaluated using the expression (*PositivePredictiveValue* + *NegativePredictiveValue* − 1), where negative predictive value is the proportion of correctly undetected regions (True Negatives) with no variants with respect to all undetected regions (including True Negatives and False Negatives). F-score and markedness of all these methods for high coverage with real data, considered in this study, are given in Table 4. A high value of F-score and markedness denotes a better validation of detected CNVs and non detected regions respectively. The value of all these measures is in [0, 1].

**Table 4:** Performance of all the compared methods using chromosome 20 data of various samples.

Sample	Method	TP	FP	FN	TN	Specificity	Precision	Sensitivity	F-Score	Markedness
NA12878	CNV-CH	340	38	112	267	0.87	0.90	0.75	0.82	0.60
	EWT	286	40	104	223	0.85	0.87	0.73	0.80	0.56
	cnMOPS	358	44	83	320	0.88	0.89	0.81	0.85	0.68
	CMDs	270	110	98	283	0.72	0.71	0.73	0.72	0.45
	CNV-TV	270	68	76	263	0.79	0.80	0.78	0.79	0.57
NA12891	CNV-CH	283	25	63	246	0.91	0.92	0.82	0.87	0.71
	EWT	348	39	85	303	0.84	0.90	0.80	0.85	0.68
	cnMOPS	303	33	99	238	0.87	0.90	0.75	0.82	0.61
	CMDS	205	93	129	170	0.64	0.68	0.61	0.65	0.25
	CNV-TV	263	51	125	190	0.78	0.83	0.67	0.75	0.44
NA12892	CNV-CH	181	21	65	138	0.86	0.89	0.73	0.81	0.57
	EWT	208	34	84	159	0.82	0.86	0.71	0.78	0.51
	cnMOPS	229	29	94	165	0.85	0.88	0.71	0.79	0.52
	CMDs	244	74	156	163	0.69	0.76	0.61	0.68	0.28
	CNV-TV	218	48	130	137	0.74	0.82	0.62	0.71	0.33
NA19238	CNV-CH	481	37	99	421	0.92	0.93	0.83	0.88	0.74
	EWT	390	94	34	451	0.83	0.80	0.92	0.86	0.74
	cnMOPS	471	75	105	442	0.86	0.86	0.81	0.84	0.67
	CMDs	159	144	106	198	0.58	0.52	0.60	0.56	0.17
	CNV-TV	348	56	87	318	0.85	0.86	0.80	0.83	0.64
NA19239	CNV-CH	529	95	122	503	0.84	0.84	0.81	0.83	0.65
	EWT	508	90	149	450	0.83	0.85	0.77	0.81	0.60
	cnMOPS	422	87	137	373	0.81	0.83	0.75	0.79	0.56
	CMDs	321	91	159	254	0.74	0.78	0.66	0.72	0.39
	CNV-TV	541	136	119	559	0.80	0.80	0.82	0.81	0.62
NA19240	CNV-CH	436	72	83	426	0.86	0.86	0.84	0.85	0.69
	EWT	424	99	154	370	0.79	0.81	0.73	0.77	0.51
	cnMOPS	490	101	176	416	0.81	0.83	0.73	0.78	0.53
	CMDs	407	129	204	333	0.72	0.76	0.66	0.71	0.38
	CNV-TV	509	136	186	460	0.77	0.79	0.73	0.76	0.50

The table represents the True Positive (TP), False Positive (FP), False Negative (FN), True Negative (TN), Specificity, Precision, Sensitivity, F-Score and Markedness values of CNV-CH and the other 4 methods, viz., EWT, cnMOPS, CMDs and CNV-TV using high coverage data considered in this study. The first column represents the sample names and the second column represents the methods against whom these values are given.

**Fig 6:**
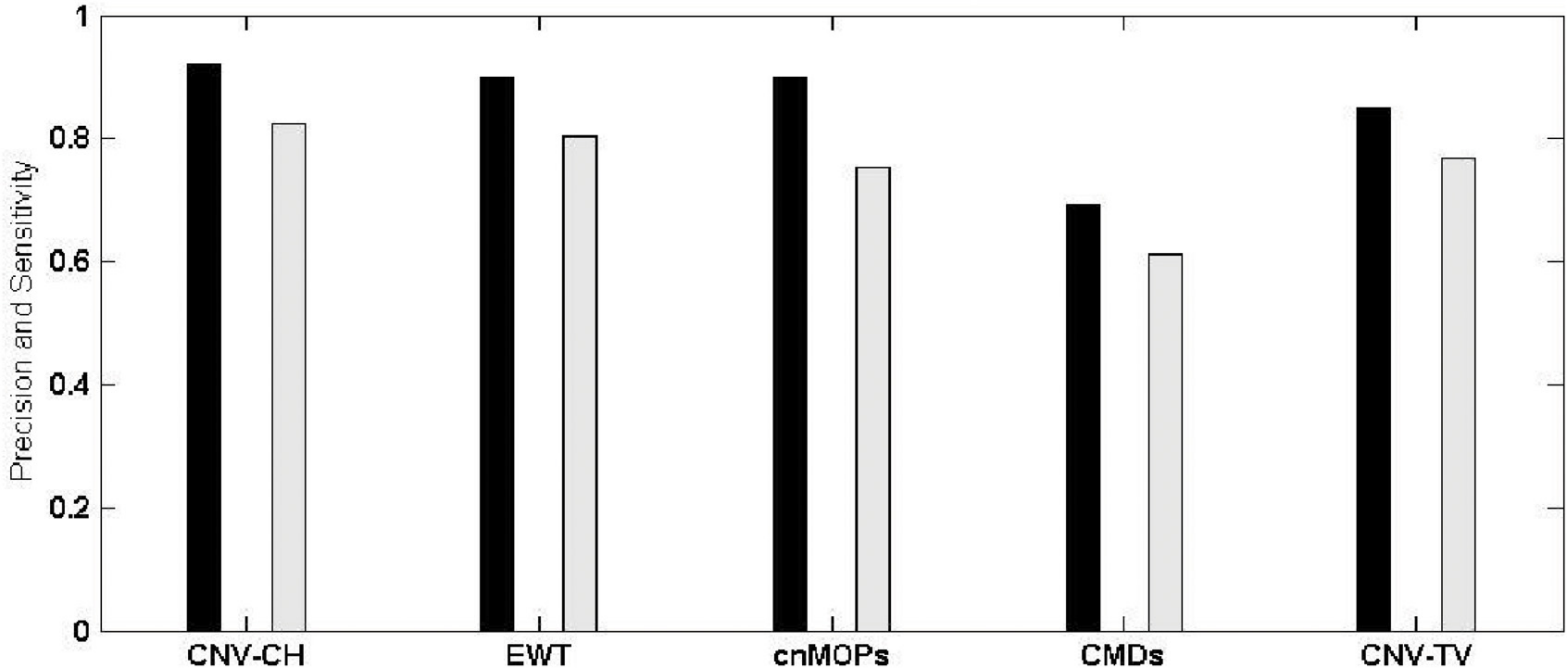
The overall precision and sensitivity of CNV-CH, EWT, cnMOPS, CMDs and CNV-TV for the sample NA12891. The black bar shows the precision value in [0, 1] and the gray bar shows the sensitivity in [0, 1].

**Fig 7:**
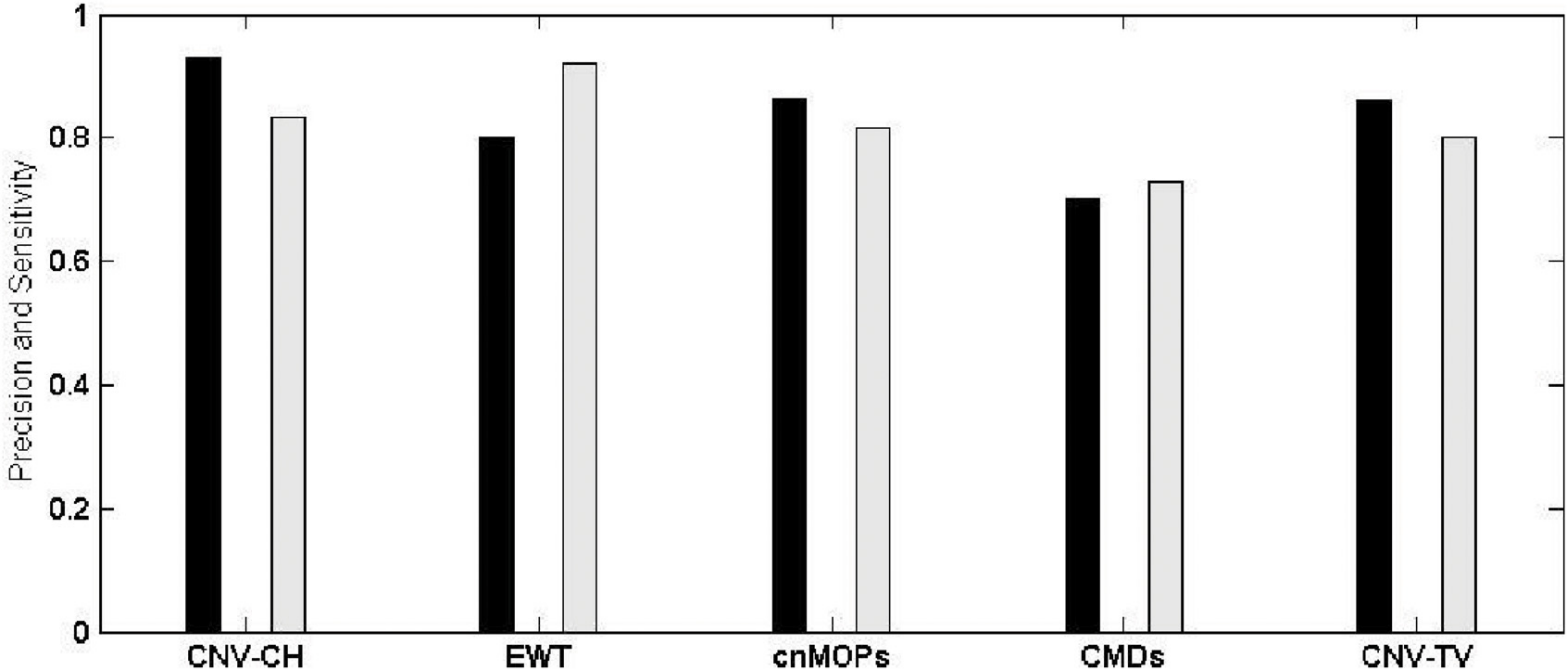
The overall precision and sensitivity of CNV-CH, EWT, cnMOPS, CMDs and CNV-TV for the sample NA19238. The black bar shows the precision value in [0, 1] and the gray bar shows the sensitivity value in [0, 1].

We considered the number of CNVs, detected by each of the above five methods, including CNV-CH, as true positives, if the genomic coordinates of CNV regions detected, has an overlap of at least 1 bp with those reported in DGV. The invalid detected regions by each method were considered as false positives. Similarly, the undetected regions which has no overlap with those reported in the DGV are considered as true negatives. Specificity, precision and sensitivity of each method were evaluated on the basis of the number of true positives, true negatives, false positives and false negatives, as found in each sample. Figs 6 and 7 show a comparative overview in terms of precision and sensitivity among these five methods for samples NA12891 and NA19238 respectively, where the former is a CEU trio male of European ancestry, and the latter is a YRI female. With reference to Fig 6, it can be observed that for sample NA12891, CNV-CH performed better than the other four methods with a higher precision and sensitivity values of 0.92 and 0.82 respectively. EWT and cnMOPS also performed well with a slightly lower precision value of 0.90. The overall performance of CNV-CH was better than the other methods as reflected in Table 4, where F-score (0.87) of CNV-CH for the sample NA12891 was higher. The method EWT showed a good overall performance with slightly lower F-score of 0.85 and cnMOPS having an F-score of 0.82. For sample NA19238, the precision of CNV-CH, as shown in Fig 7, was higher with a value of 0.93 than that of the other methods. But for this sample, EWT performed better in terms of sensitivity with a value of 0.92. The sensitivity of CNV-CH for the sample NA19238 was observed to be 0.83, which is reasonably good with a higher precision value. Here, both cnMOPS and CNV-TV also obtained good sensitivity values of 0.81 and 0.80 respectively. The method CMDs performed poorly with respect to both sensitivity and precision in all the samples, as compared to the other methods. It can also be observed from Table 4 that CNV-CH outperformed all the other methods, in terms of specificity and markedness, with an exception for the sample NA12878, where cnMOPS obtained better results. In sample NA12878, cnMOPS outperformed CNV-CH marginally, with respect to specificity and markedness, as shown in the table.

**Fig 8:**
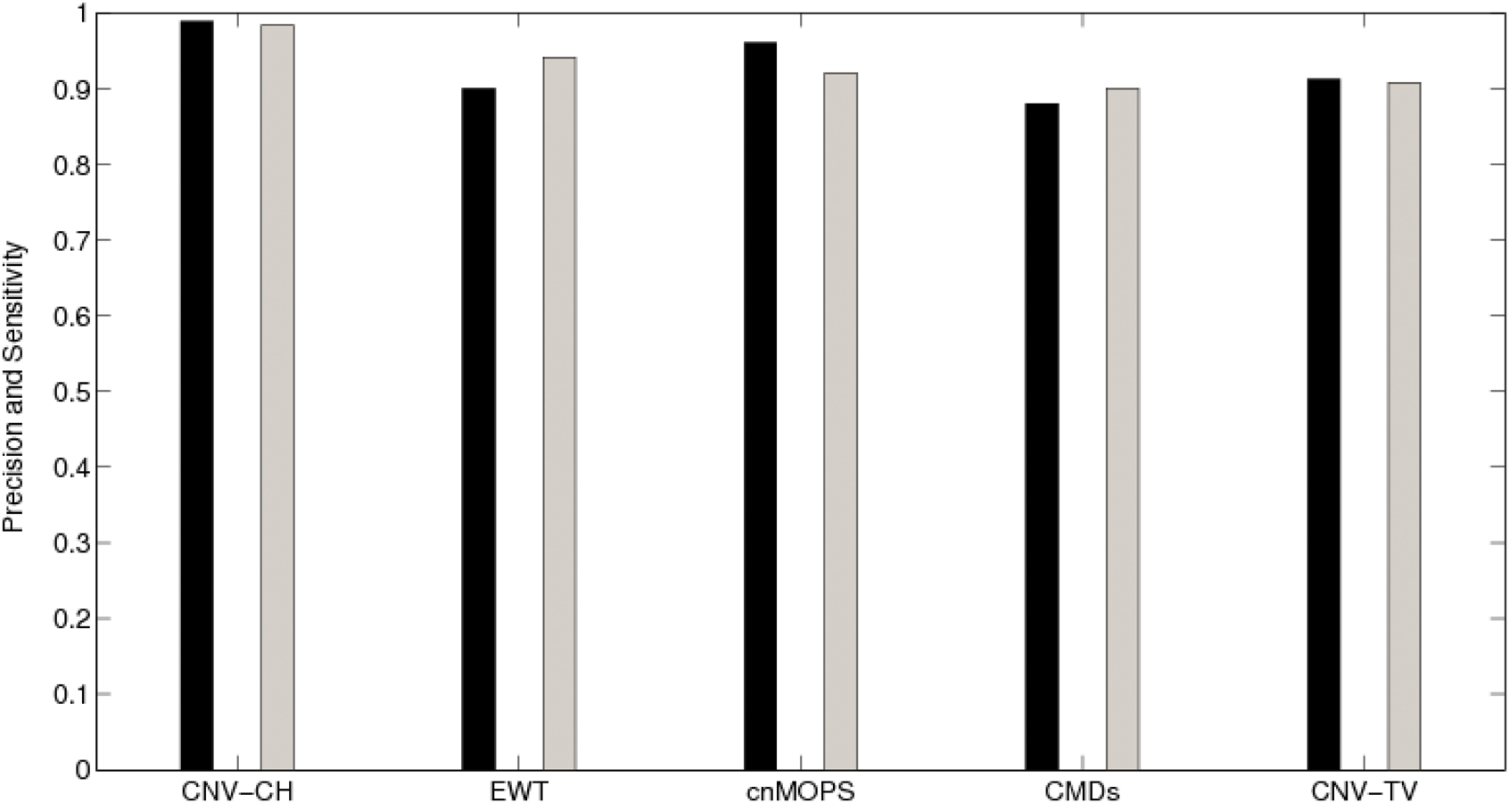
The overall precision and sensitivity of CNV-CH, EWT, cnMOPS, CMDs and CNV-TV on the basis of their performance on simulated data set. The black bar shows the precision value in [0, 1] and the gray bar shows the sensitivity value in [0, 1].

**Fig 9:**
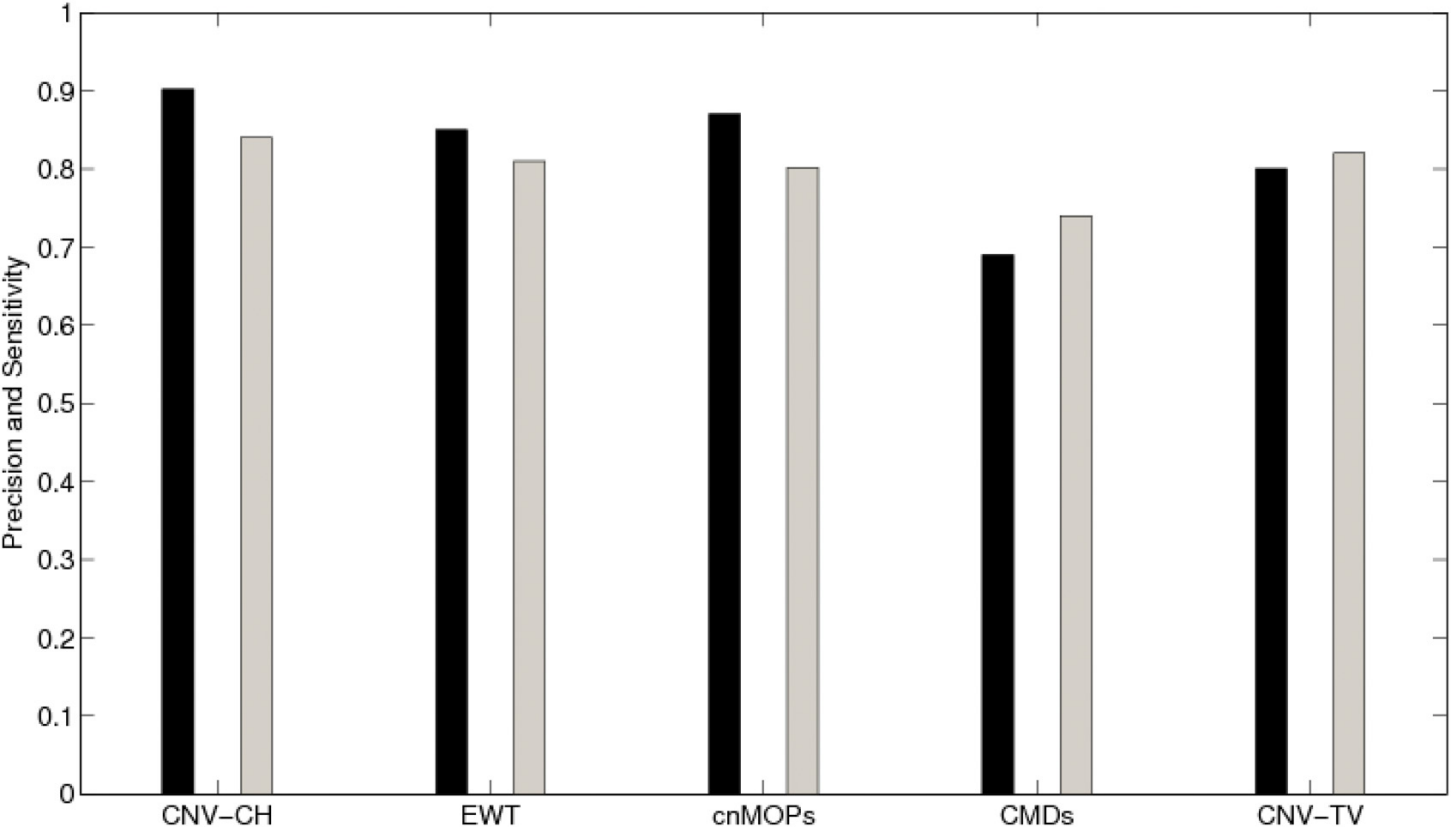
The overall precision and sensitivity of CNV-CH, EWT, cnMOPS, CMDs and CNV-TV, on the basis of their performance on the real data set considered in our work. The black bar shows the precision value in [0, 1] and the gray bar shows the sensitivity value in [0, 1].

We also compared the performance of the above five algorithms on the simulated data set, described in Result section. We observed that almost all these methods had performed well on this data set, as shown in Fig 8. However, CNV-CH outperformed all with a very high precision, sensitivity, and F-score values of 0.98 each. Apart from CNV-CH, the sensitivity of EWT was higher than the other methods with a value 0.94. cnMOPS also performed quite better for our simulated datasets, with a high precision and sensitivity values of 0.96 and 0.92 respectively (Fig 8). Here, the performance of CMDs and CNV-TV was relatively poor, in terms of both precision and sensitivity, as observed in the figure. Similarly, Fig 9 depicts the performance of the above algorithms, including CNV-CH, on a real data set described in Materials and Methods section.

## Discussion

Here we have developed an algorithm, called CNV-CH, for detecting copy number variation in genomic locations, using next generation sequencing data. CNV-CH involves a novel segmentation approach to detect genomic regions having copy number variations, across multiple samples, based on the notion of a convex hull algorithm.

Reads generated from repeat rich regions suffer from mappability bias which may lead to false detection of variants. To deal with this issue, CNV-CH considers all possible *k*–mers from the reference DNA sequence, where *k* is the minimum length of a read. These *k*–mers were aligned to the same reference sequence for calculating the mappability score of each base position. The mappability score corresponding to a base position is the ratio of total number of *k*–mers aligned at that base position uniquely and the total number of *k*–mers containing that base position [20]. Hence, repeat rich region will have a smaller mappability score. Read counts corresponding to bins having a lower mappability score were adjusted by performing exponential smoothing, where the factor *α* is set to the mean mappability score of that bin. CNV-CH analyzes the depth of coverage data in a novel manner, by transforming each of the GC-corrected read count data into a two dimensional geometrical point, where the first dimension is obtained by converting the read count data into a standardized normal score and the other dimension is obtained by representing the read count data with the cumulative binomial distribution function. Another novelty of our work is the deployment of a new segmentation algorithm across all the samples, where the notion of convex hull is used to identify the clusters of consecutive bins, depicting copy number variations.

CNV-CH has analyzed the real sequencing data of chromosome 20 of multiple human individuals. These human genome sequences are based on deep coverage whole genome sequencing data. Each detected region was validated with the variants listed in the Database of Genomic Variants (DGV). CNV-CH detects both common and rare CNVs with equal computational efficiency. For the samples, the CNV regions, detected by our method, were mostly greater than 10 kbp in length. Moreover, our algorithm also detected CNV regions as small as 0.6 kbp. We also performed a simulation study by considering a real human DNA sequence of chromosome 20 and processing it to generate the control genome and *n* test samples. This simulation study was performed by varying the following experimental conditions, viz., Coverage, Variant length, Copy number, Number of test samples and Position represents the genomic coordinate where a copy variation has been introduced. We considered all possible experimental settings, where each condition was a combination of the above values. We observed that CNV-CH performed efficiently in detecting the start and end locus of the segment with variation, at the base pair level, where a very high precision, sensitivity and F-score values of 0.98 (each), were achieved.

We compared the performance of CNV-CH with four existing algorithms- EWT, CNV-TV, CMDs and cnMOPS, used for analysis of copy number variations. The approach of cross sample analysis, adopted by CNV-CH, achieved high precision value due to low false positive detections. The percentage of overlap of CNV regions, detected by the four existing algorithms with those detected by CNV-CH, was analyzed. It was observed that the validated regions detected by our algorithm overlapped maximum with EWT. The overall performance of our method was better than the others, with a high precision value in [0.89, 0.94], and reasonably good sensitivity values in [0.71, 0.83] for all the real test samples. The quality of the output of our algorithm was also evaluated and analyzed using other standard measures, like specificity, F-score and markedness. CNV-CH resulted in higher F-score values compared to the other existing algorithms, in [0.81, 0.88] and markedness varies in [0.57, 0.74]. To our knowledge, most of the existing algorithms assume the read count data to follow a particular statistical distribution. On the other hand, we have considered the distribution of read count data to follow two different distribution models independently. It adds robustness against the error that may arise from incorrect assumption of a particular statistical distribution model. Moreover, the present algorithm also detects CNV breakpoints with higher efficiency.

As CNV-CH processes multiple human genome sequence data obtained through NGS technology, it involves high computational resource in terms of memory. We implemented our algorithm using a 64 bit machine with 16 GB RAM. As our method is using the notion of convex hull, which requires at least 3 non-collinear points, thus algorithmically, the minimum number of samples required is 3. The complexity of the algorithm was found to be *O*(*nklogk*), where *k* is the total number of samples and *n* is the number of 100bp bins. As cross sample analysis has been found to produce effective results with 6–10 samples, hence the complexity *O*(*nklogk*) is almost equal to *O*(*n*) as *klogk* can be approximated as a constant term. However, if outlier data occur too frequently in different samples over several consecutive bins, the performance of CNV-CH will degrade. As a solution to this problem, removal of the outlier data in the preprocessing step will significantly improve the performance of the present algorithm. Further work needs to be done for detection of outliers and their removal.

## Materials and Methods

The present method CNV-CH is based on a novel approach that has considered the read depth information of multiple samples as two dimensional geometric points, where each dimension is generated using a statistical measure. Next important contribution of our method is the segmentation of the genomic region. It is performed based on the idea of creating convex hull of a geometric region, formed by the previously generated two dimensional points. Next, genomic segments, having copy number changes, are detected based on the area of the convex hull thus created. Our method has also considered the presence of outlier data separately. The series of processes that CNV-CH goes through are detailed below.

### Input data and its processing

Let us consider *n* samples obtained by using next generation sequencing technology. Each sample is associated with millions of short reads generated from a DNA sequence in a particular chromosome. These reads are present in a sequence alignment file (.sam file) or in the form of a compressed binary file (.bam file), and are maintained in different sequence databases as discussed in Datasets considered in the present study section. Let us consider a standard reference DNA sequence of the same chromosome.

#### Bins consideration

We considered a standard approach of partitioning the reference genome into *w* non-overlapping bins. For each individual sample, the reads were mapped to the bins of the standard reference sequence. This mapping was done using Burrow Wheeler alignment algorithm [21], allowing 3 mismatches. If a read maps to multiple locations, then it is mapped to any one location randomly, i.e., a read will map to a single location only. This mapping was done for all these *n* samples separately. Now for each sample, the number of reads getting mapped to a bin of the reference sequence, was counted (read count). In this way, for all *w* bins, the read counts were found out. Hence, by the above process, we got *w* read counts for each of these *n* samples. The choice of non-overlapping bin was made because, in our work, the read count (read depth) was calculated on the basis of many-to-one mapping (aligning) of reads to 100bp bins of the reference. In other words, a particular read has been aligned to one particular bin. In case of overlapping bins, almost every read would have a possibility of multiple optimal alignment, resulting in higher ambiguity in mapping or alignment of most of the reads, which would incur higher mappability bias in read depth data, as compared to non-overlapping bins. This higher mappability bias in read count data of adjacent overlapping bins would cause abrupt variations in read count data of two consecutive overlapping bins, even in regions without genomic variation. Here, the size of non-overlapping bins was chosen as 100 bp each, for getting higher accuracy in detecting CNV breakpoints [13], with an assumption that the CNV, spanning partially in a 100bp bin, will have an error between 0 and 100bp only.

#### Adjustment of GC and Mappability bias

The read counts suffer from GC-content bias [13, 22] and need to be adjusted using the following formula,

$$AdjustedReadCount=u_{ij}\times \left(d_{i}/d_{GC}^{ij}\right),$$ (1)

where *u*_*ij*_ is the number of reads (read count) in *i*th sample mapped to *j*th bin, *d*_*i*_ is the median read count of *i*th sample across all the bins, and $d_{GC}^{ij}$ is the median read count of those bins which have the same GC-content as *j*th bin of *i*th sample.

Reads generated from repeat rich regions cannot be mapped uniquely to a particular bin. These reads get randomly aligned to one of the bins, with an optimal alignment score. Thus, the resultant read count data of the bins, corresponding to repeat rich regions of the genome, will suffer from biases (high or low). This mappability bias may lead to false detection of variants. In order to deal with this issue, all possible *k*–mers from the reference DNA sequence are generated, where *k* is the minimum length of a read. These *k*–mers were aligned to the same reference sequence for calculating the mappability score of each base position. It is to be mentioned that the mappability score corresponding to a base position is the ratio of total number of *k*–mers aligned at that base position uniquely and the total number of *k*–mers containing that base position [20]. Now, the mappability score of each bin is calculated as the mean mappability score over all the 100 bases in the bin. The mappability score of repeat rich regions will be low, and hence read counts corresponding to bins having a lower mappability score are adjusted, by performing exponential smoothing, where the factor *α* is set to mean mappability score of that bin.

Finally, we have obtained a two dimensional matrix **R** of order *n* × *w*, containing adjusted read counts of *n* samples across *w* non-overlapping bins of size 100bp each. Each entry *r*_*ij*_ of **R** denotes modified read count of *i*th sample mapped to *j*th bin of the reference genome.

### Transformation of read count information into two dimensional geometric points using statistical measures

As mentioned in the Introduction section, copy number variation occurs due to deletions, duplications or insertions in genomic regions. If a sample has a duplication, insertion or deletion in a particular region, the bins of the reference sequence belonging to these regions will have a significantly high or low read counts respectively. CNV-CH treats the input matrix **R** in a novel manner by transforming each element into a 2*D* geometric point, so that the convex hull can be obtained for segmentation as described later. Now, the transformation of *r*_*ij*_ is done using the following statistical measures.

- **Standardized score of read counts:** Read count *r*_*ij*_ is not standard across all samples, as the chemical process of generating reads for each sample is done independently and sometimes read generation also suffers from low quality sequencing. Hence a standardized score of read count needs to be adopted for establishing uniformity across all the samples. The standardized read count, denoted by *zrow*_*ij*_ [23], is obtained by
  $$zrow_{ij}=\frac{\left(r_{ij}-\mu_{i}\right)}{\sigma_{i}},$$ (2)
  where 1 ≤ *i* ≤ *n* and 1 ≤ *j* ≤ *w*, and *μ*_*i*_ is the mean read count of *i*th sample and *σ*_*i*_ is the standard deviation of read count of the *i*th sample over all the bins. When read count *r*_*ij*_ is high, *zrow*_*ij*_ value will be high and vice versa. Thus the regions having structural variation will have very high or very low read count corresponding to duplication, insertion or deletion, and hence *zrow*_*ij*_ value will also be very high or low.
- **Cumulative distribution function of read counts:** In order to find out the read count data, we consider set of reads that are to be mapped against the non-overlapping bins of the reference sequence. If we consider a particular bin, we observe that out of the total reads available, only a few will get aligned to the bin. Here, mapping of a read to a bin is called a trial. The trials in which the reads get successfully aligned to a bin, are termed as “successes”, and the remaining trials correspond to “failures”. This kind of the alignment process leads us to consider the read count data over all the bins to follow a binomial distribution. It has motivated us to represent the read count data with the cumulative binomial distribution function. We have calculated the value of the cumulative distribution function for each read count (*r*_*ij*_) of *i*th sample getting mapped to *j*th bin on the basis of binomial distribution [23]. Here, the reads (*r*_*ij*_) getting mapped to *j*th bin are considered as *successes* (successful alignment) and the remaining (*t*_*i*_ − *r*_*ij*_) reads as *failures*, *t*_*i*_ being the total number of reads of *i*th sample. Hence, if *p* is the probability of a read getting successfully mapped to a bin, then the probability of *r*_*ij*_ successes is given by
  $$P\left(r_{ij}\right)={}^{t_{i}}C_{r_{ij}}p^{r_{ij}}q^{\left(t_{i}-r_{ij}\right)}$$ (3)
  where *q* = 1 − *p*, i.e., probability of failure (failed alignment). Therefore, the cumulative distribution function (CDF) [23] of *r*_*ij*_ is
  $$cf_{ij}=\sum_{k=0}^{r_{ij}}{}^{t_{i}}C_{k}p^{k}q^{t_{i}-k}$$ (4)
  Here, we have obtained, on the basis of maximum likelihood estimation [23], the value *p* as 1/*w*, where *w* is the total number of non-overlapping bins.

Thus, we have used a novel technique to represent each *r*_*ij*_ by a two dimensional point where the first dimension is represented by *zrow*_*ij*_, while the other one by *cf*_*ij*_. The cluster of consecutive bins having high or low read counts, due to duplications, insertion or deletions will correspondingly depict high or low *zrow*_*ij*_ as well as *cf*_*ij*_ values. This novel way of representation of read counts as two dimensional points (*zrow*_*ij*_, *cf*_*ij*_) adds robustness in the process of CNV analysis. Finally, we have got a 2–dimensional array **G**_*n*×*w*_, where each **g**_*ij*_ entry is a vector denoted by [*zrow*_*ij*_, *cf*_*ij*_]^*T*^, 1 ≤ *i* ≤ *n*, 1 ≤ *j* ≤ *w*.

### Segmentation over multiple samples

Insertions, deletions or duplications, causing copy number variations, are normally of size greater than 1kbp and smaller variations are called indels. Since we have taken a bin size of 100bp, the genomic regions with a CNV will span across multiple consecutive bins. Our method uses a novel technique to perform segmentation of genomic regions considering the notion of convex hull, to identify the clusters of consecutive bins, depicting copy number variations. For segmentation, we have obtained convex hull over the previously obtained **G**_*n*×*w*_ array. Convex hull is a convex polygon enclosing a set of geometrical points having minimum area [24]. Genomic regions (bins) having similar copy numbers will tend to have similar standardized values of *zrow*_*ij*_ and *cf*_*ij*_, and hence the two dimensional points **g**_*ij*_ corresponding to these bins, will be in close proximity to each other in geometric space. Therefore, a convex hull enclosing these similar geometric points will have a small area. Based on this notion of geometric orientation of points (of **G**), we have obtained convex hull to identify segmentation of the bins and extract the region having some variations. The algorithm below describes the process of creating convex hull applied over **g**_*ij*_ points of **G** array, and also describes how segmentation of the bins is done to detect regions (consecutive bins) having CNV.

Input: **G**_*n*×*w*_ array.
Output: Genomic locations with copy number variations.
Steps:
  1. Initialize cnvflag = false, which implies that there is no genomic variation across the region. The flag will become true if an abnormal genomic region is encountered.
  2. For each bin *j* from 1 to *w* − 1 do:
    - Create a convex hull using points of consecutive *j*th and *j* + 1th bins (column of the array **G**_*n*×*w*_), where 1 ≤ *j* < *w*, across *n* samples (*i.e*., row of array **G**_*n*×*w*_). These points with which a convex hull is created are **g**_*ik*_, 1 ≤ *i* ≤ *n*, *j* ≤ *k* ≤ *j* + 1. Let the area of this convex hull obtained be *A*_*jl*_, where *l* = *j* + 1.
    - Create a second convex hull using a set of points of *j*th bin only. The points are denoted by **g**_*ij*_, 1 ≤ *i* ≤ *n*. Let the area of this convex hull be *a*_*j*_.
    - Create a third convex hull using the set of points of (*j* + 1)th bin only. The points of this bin are denoted by **g**_*il*_, 1 ≤ *i* ≤ *n*, *l* = *j* + 1. Let the area of this convex hull be *a*_*l*_.
    - Calculate △_*jl*_ = 2*A*_*jl*_ − (*a*_*j*_ + *a*_*l*_), *l* = *j* + 1.
    - If △_*jl*_ is significantly high (> *θ*) and cnvflag = false then (It implies a breakpoint from normal to an abnormal genomic region.)
      - Mark bin *l* = *j* + 1 as the starting boundary of an abnormal genomic location. Let the notation for starting bin or boundary be *stb* = *l* = *j* + 1;
      - Set cnvflag = true;
      - Set cnvlength = 1;
    - If cnvflag = true and △_*jl*_ is significantly high (> *θ*) then (It implies a breakpoint from abnormal genomic region to a normal region.)
      - Mark bin *j* as the end boundary of the abnormal genomic region. Let the notation for the end bin or boundary be *enb* = *j*;
      - Reset cnvflag = false;
      - If length of detected region, *i.e*., (*enb* − *stb* + 1) ≥ 5 then (It implies that the detected region is at least 500*bp*.) Record the detected region as a potential CNV region.

In the above segmentation algorithm the expression

$${△}_{jl}=2A_{jl}-\left(a_{j}+a_{l}\right),$$ (5)

will have low value if the consecutive bins belong to similar genomic regions, irrespective of whether the region is normal or affected by CNV. The value of △_*jl*_ will be high at breakpoints where the consecutive bins will have different genomic structures. Now precisely identifying the actual locus or base will be of concern in the starting and ending bin, which is solved by left to right scan for finding the rightmost segment within the start bin, with the most significantly high or low (gain or loss) value of the segment’s average base coverage, with respect to mean read count of that sample. The starting locus of this segment will give the start base position of the CNV region. Similarly, the end base position of the ending bin of the sample is also detected by finding the leftmost segment within the end bin, with the most significantly high or low (gain or loss) value of the segment’s average base coverage with respect to mean read count of that sample. Now, the copy number is estimated by using the expression 2*r*_*ij*_/*μ*_*i*_ as discussed in [13].

#### Presence of Outlier

Another important issue is the presence of outlier in the read count data. The presence of an outlier in read count data, in any one of the bins of a similar genomic segment, will affect the process of segmentation. The presence of such outlier in a bin will make the bin dissimilar to its immediate preceding as well as its immediate succeeding bins. Thus, the value of △_*v*−1,*v*_ of Eq (5) will be high, where *v* is the bin containing outlier data. This high value of △_*v*−1,*v*_ will be treated as a breakpoint. Similar situation arises with △_*v*,*v*+1_ value, if *v*th bin contains outlier data. This high value of △_*v*,*v*+1_ will be treated as another breakpoint. Thus, the gap between these two breakpoints comprises only a single bin. If the bin containing outlier data lies in a normal genomic region, the above breakpoints will give rise to a CNV region of size 1 bin. This CNV region with 1 bin will automatically be ignored by our algorithm. If the bin, containing outlier data lies in an abnormal genomic region, the breakpoints will incorrectly divide the segment into two subsegments. Hence, to avoid such subdivision, merging of bins is required. In other words, the method will perform merging of two consecutive subsegments, if they are separated by one bin, i.e., the value of △_*x*,*y*_ (in Eq 5) will be low, where *x* denotes the end bin of one subsegment, *y* is the start bin of other consecutive subsegment, and *y* − *x* = 1.

The performance of the present algorithm will degrade if outlier data occur too frequently in different samples over several consecutive bins. As a solution to this problem, removal of the outlier data in the preprocessing step will significantly improve the performance of the present algorithm. Further work needs to be done for outlier detection and its removal. Moreover, as our method is using the notion of the convex hull, which requires at least 3 non-collinear points, the minimum number of samples required is 3.

## Supporting Information

S1 Table All the regions with variations, detected by CNV-CH, throughout human chromosome 20. The table includes data for both high and low coverage samples.
(XLSX)
